# Comparison of Standard and Transepithelial Corneal Cross-Linking for the Treatment of Keratoconus: A Meta-analysis

**DOI:** 10.1155/2021/6679770

**Published:** 2021-01-29

**Authors:** Yu Di, Jingyi Wang, Ying Li, Yang Jiang

**Affiliations:** Department of Ophthalmology, Peking Union Medical College Hospital, Chinese Academy of Medical Sciences, Beijing 100730, China

## Abstract

**Purpose:**

To compare the clinical results of standard corneal cross-linking (SCXL) with transepithelial corneal cross-linking (TECXL) in progressive keratoconus using a meta-analysis.

**Methods:**

PubMed, EMBASE, and Cochrane Central Register of Controlled Trials were searched up to June 2020 to identify relevant studies. The PRISMA guidelines were followed. Primary outcomes were change in uncorrected distance visual acuity and maximum keratometry (*K*_max_) after CXL. Secondary outcomes were change in corrected distance visual acuity, mean refractive spherical equivalent (MRSE), spherical and cylindrical error, endothelial cells density (ECD), and central corneal thickness (CCT).

**Results:**

Sixteen studies with a total of 690 eyes (SCXL: 332 eyes; TECXL: 358 eyes) were included. At the last follow-up, SCXL provided a greater decrease in maximum keratometry (*K*_max_) than TECXL (weighted mean difference (WMD) −1.12; 95% confidence interval (CI) −1.96, −0.29). For the other outcomes, there were no statistically significant differences.

**Conclusions:**

Except for a greater decrease in Kmax with SCXL group, both groups have a comparable effect on visual, pachymetric, and endothelial parameters at 24 months after surgery. Larger studies with a longer follow-up time are necessary to determine whether these techniques are comparable in the long term.

## 1. Background

Keratoconus is a progressive, noninflammatory corneal degeneration that leads to corneal thinning, myopia, irregular astigmatism, and reduced visual acuity. The disorder often begins in the second decade of life and always affects both eyes, albeit sometimes to highly varying degrees [1]. A recent meta-analysis performed over seven million patients from 15 different countries has calculated a global prevalence of 138/100,000 [2]. Early in the disease, when affected individuals experience the first symptom, changes in corneal refractive power can generally be corrected with glasses. As astigmatism becomes increasingly irregular, special dimensionally rigid contact lenses are mostly need to be used. If, eventually, contact lenses can no longer be fitted, corneal transplantation may become necessary for the purpose of visual rehabilitation [3]. In the recent two decades since the cornea cross-linking (CXL) was introduced, multiple prospective studies have firmly established its role as an integral aspect of the management of early keratoconus to tackle the previously unaddressed component of halting the ectatic process [4].

CXL was first introduced by Wollensak et al. [5] as a promising technique to slow or stop the progression of keratoconus. It uses the photochemical interaction of ultraviolet A radiation (UVA) and riboflavin (vitamin B2) to induce cross-linking between corneal stromal macromolecules, resulting in increased biomechanical stiffness and improved resistance to enzymatic digestion [6]. The standard corneal cross-linking (SCXL) uses a 3 mW/cm^2^ UVA intensity for 30 min irradiation time. Many studies have reported on the safety and efficacy of SCXL in the treatment of keratoconus patients [7–9]. Shajari et al. performed a meta-analysis, supporting the efficacy of SCXL [10]. However, it also has some drawbacks, such as pain, discomfort, delays visual rehabilitation, and infection [9]. Recently, transepithelial corneal cross-linking (TECXL) has been introduced. It is a novel modification of SCXL that maintains the integrity of the corneal epithelial layer and has a higher UVA intensity, allowing patients a faster recovery after a more comfortable treatment process [11, 12]. Ameerh et al. [13] proposed that TECXL might be an effective method in halting the progression of keratoconus without the side effects of epithelial removal, which can be encountered in the SCXL procedure.

Some studies have compared postoperative outcomes between the SCXL and TECXL in keratoconus patients, but conclusions lack consistency [13–15]. The study performed by Akbar et al. [14] demonstrated that SCXL and TECXL have a comparative effect on UCVA and CDVA at one year after surgery. However, Cifariello et al. [15] proposed that TECXL is preferable to SCXL since it preserves the corneal thickness and improves visual acuity at 24-month follow-up. Bikbova et al. [16] found that stabilization and regression of keratometry values were achieved in both groups, but SCXL was more effective at 24-month follow-up. The discrepancy might be related to the follow-up time.

To our knowledge, no meta-analysis has discussed the efficacy and safety results of SCXL versus TECXL in keratoconus patients at different follow-up times after surgery. Therefore, our goal was to compare outcome changes of SCXL with TECXL in the treatment of keratoconus at three-, six-, twelve-, or twenty-four-month follow-up.

## 2. Methods

This meta-analysis was performed according to the guideline given by the Preferred Reporting Items for Systematic Reviews and Meta-analysis (the PRISMA statement) [17].

### 2.1. Search Strategy

The PubMed, Embase, and Cochrane Central Register of Controlled Trials were searched from their earliest entries thorough June 2020, to identify studies potentially eligible for this review. The following key words or corresponding medical subject headings (MESH) were used: “keratoconus,” “cross linking,” “transepithelial,” and “epithelial-on”. The detailed search criteria were ((“Keratoconus” [MeSH Terms] OR (“Keratoconus” [Title/Abstract] OR “keratoconic” [Title/Abstract])) AND ((((“cross-link” [Title/Abstract] OR “cross-link*∗*” [Title/Abstract]) OR “cross link” [Title/Abstract]) OR “cross-link*∗*” [Title/Abstract]) OR “cross-linking” [Title/Abstract])) AND (((“transepithelial” [Title/Abstract] OR “trans-epithelial” [Title/Abstract]) OR “epithelial-on” [Title/Abstract]) OR “epi-on” [Title/Abstract]). The reference lists of the relevant articles were also manually examined to identify additional potentially related studies. No language restriction was imposed.

### 2.2. Inclusion and Exclusion Criteria

Inclusion criteria were as follows: (1) participants: a patient (age ≥ 18 years) with keratoconus; (2) intervention: CXL; (3) comparison: SCXL versus TECXL; (4) studies containing one of the following outcomes: changes in uncorrected distance visual acuity (UDVA), maximum keratometry (*K*_max_), corrected distance visual acuity (CDVA), mean refractive spherical equivalent (MRSE), spherical and cylindrical error, endothelial cells density (ECD), and central corneal thickness (CCT) at three, six, twelve, or twenty-four months after CXL.

Exclusion criteria were as follows: (1) studies that examine only one CXL modality (transepithelial or transepithelial-off) without a comparator; (2) studies that examine CXL combined with other treatments (photorefractive keratectomy or intrastromal corneal ring segments); (3) animal studies or cadaver subjects; (4) reviews, case reports, correspondence, conference presentations, and unpublished data.

### 2.3. Outcome Measures

Primary outcomes were change in UDVA and Kmax after CXL. Secondary outcomes were change in CDVA, MRSE, spherical and cylindrical error, ECD, and CCT.

### 2.4. Data Extraction and Assessment of Methodological Quality

After achieving the potentially relevant articles, the EndNote software was used to remove the duplicates. Then, the title and abstracts of the remaining articles were reviewed to filter the unrelated studies. The next step was to obtain the full texts of each article and review them; the articles that met the eligibility criteria and failed the exclusion criteria were included. The studies were reviewed by two authors (D.Y. and W.J.Y) independently. Data extracted included the first author's name, publication year, study location, design, number of eyes, mean age, follow-up time, details of treatment protocols, and evaluated variables at different follow-up time points.

The Cochrane Collaboration tool was applied to evaluate the risk of bias of randomized controlled trails (RCTs) [18]. Evaluation was conducted in terms of random sequence generation and allocation concealment (selection bias), blinding of participants and personnel (performance bias), blinding of outcome assessment (detection bias), incomplete outcome data (attribution bias), selective reporting (reporting bias), and other biases by grading with low, high, or unclear risk for the study. For nonrandomized comparative studies, the Newcastle-Ottawa Scale (NOS) was applied [19]. The NOS includes study object selection (four items and four points), intergroup comparability (one item and two points), and the results of measurement (three items and three points), with a total score of nine points. Studies with more than seven points can be considered of a high quality. Each study was independently assessed by two authors (D.Y and W.J.Y). Discrepancies were reconciled by discussing with the corresponding author (L.Y).

### 2.5. Statistical Methods

Statistical analyses were performed with Review Manager (REVMAN, Version 5.3). Treatment effects were evaluated as a weighted mean difference (WMD) and 95% confidence interval (CI) calculated for the absolute change of the interested outcomes. For individual articles, WMD was computed by the difference of the mean change in the SCXL group and that in the TECXL group. The outcomes were measured as mean ± standard deviation. Heterogeneity between studies was determined using the chi-square test and by computing the quantity *I*^2^ statistics. An *I*^2^ greater than 50% was considered to state significant heterogeneity. Random-effect models were used since studies were assumed to differ from each other regarding the aspects of implementation.

Publication bias was assessed by Begg's funnel plot and Egger's leaner regression teat. The analyses were performed using StataSE (Version 15.1). A *P* < 0.05 was considered statistically significant.

## 3. Results

### 3.1. Study Characteristics

**Figure 1:**
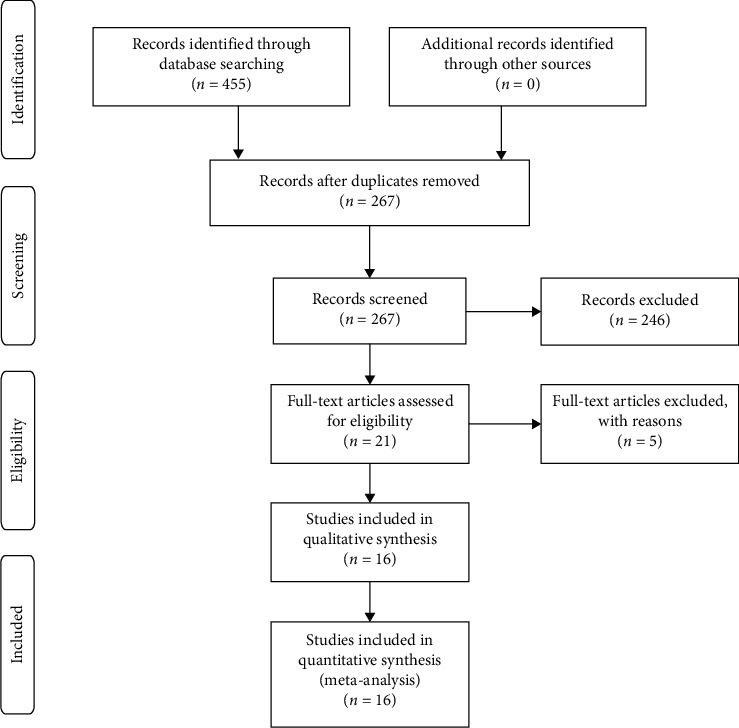
Flowchart depicting the selection of included studies.

**Figure 2:**
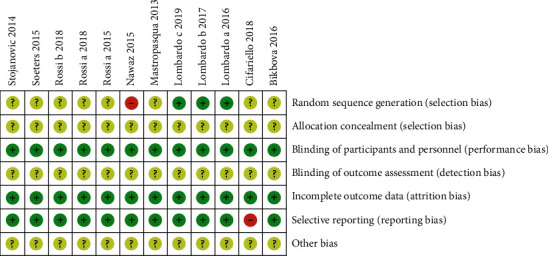
Risk of bias of randomized controlled trials included in the meta-analysis.

A total of 455 potentially relevant articles were identified for the meta-analysis. After removal of duplicates (*n* = 188) and screening of titles and abstracts (*n* = 267), 21 potential articles were assessed for eligibility. After reviewing full-text and applying inclusion and exclusion criteria, three studies were excluded for not meeting the inclusion conditions, and one study was also excluded because of the incomplete datasets. Two studies were published based on a clinical trial performed at the University Medical Center Utrecht in the Netherlands. We selected one study because it included more measurements than the other [20]. Therefore, 16 studies qualified for the meta-analysis (Figure 1) [15, 16, 20–33]. Rossi et al. [31] evaluated the outcomes of conventional Drusen protocol compared with two different protocols of TECXL. We compared the standard group with one particular transepithelial group, respectively, and treated the whole study as if there had been two single trials. However, when calculating the number of eyes in both groups, the eyes of the SCXL group were only included once. Lombardo et al. published three articles reporting the results of six-, twelve-, and twenty-four-month follow-up from a study conducted at the Fondazione G.B. Bietti's clinical center (Rome, Italy) [24–26]. We included the three articles, but the eyes were included only once in the calculation of the total number of eyes. Therefore, a total of 332 eyes were treated with SCXL and 358 eyes underwent TECXL. In addition, among 16 studies, four studies reported 24-month follow-up results. The 24-month data are available at most in 145 eyes (43.7%) of the total eyes with SCXL and 158 eyes (44.1%) of the total eyes with TECXL. Characteristics of all the studies and details of the treatment protocols are presented in Table 1. The methodological quality of the RCT has been shown in Figure 2. Most of the trails seemed to not pay enough attention to the aspects of “random sequence generation and allocation concealment,” “blinding of outcome assessment,” and “other bias.” Except for those previously mentioned, the results of the other aspects were satisfying.  Table 2 has shown the risk of bias assessment based on the NOS for non-RCT, and the total scores of all five trails were not lower than seven points.

### 3.2. Primary Outcomes

#### 3.2.1. Uncorrected Distance Visual Acuity

**Figure 3:**
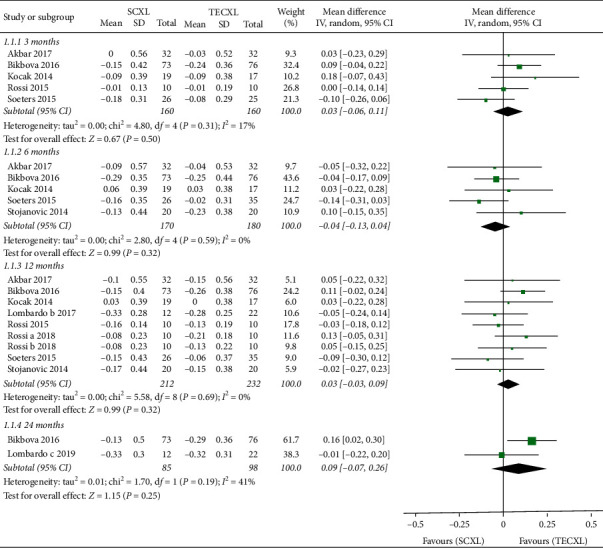
Changes in uncorrected distance visual acuity in standard corneal cross-linking (SCXL) and transepithelial corneal cross-linking (TECXL). CI = confidence interval; IV = inverse variance.

The change in UDVA did not significantly differ between the two groups through the follow-up (*P* = 0.50, 0.32, 0.32, and 0.25, resp.) as provided in the forest plot, but TECXL seems to improve UDVA more than SCXL at 12- and 24-month follow-up (*P* = 0.32 and 0.25) (Figure 3).

#### 3.2.2. Maximum Keratometry

**Figure 4:**
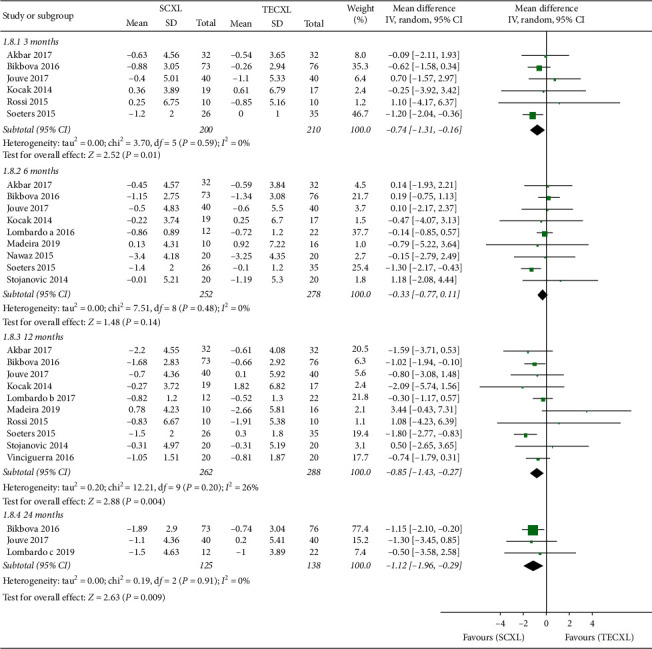
Changes in maximum keratometry in standard corneal cross-linking (SCXL) and transepithelial corneal cross-linking (TECXL). CI = confidence interval; IV = inverse variance.

For the change in Kmax, SCXL provided a greater decrease throughout the follow-up (*P* = 0.01, 0.14, 0.004, and 0.009, resp.) (Figure 4).

### 3.3. Secondary Outcomes

#### 3.3.1. Corrected Distance Visual Acuity

At the early three-month visit, the improvement in CDVA was comparable in both groups (*P* = 0.51). At the six- and twelve-month visits, TECXL showed better results (*P* = 0.11 and *P* < 0.00001), but at the 24-month visit, there was no difference in CDVA between the two groups (*P* = 0.97) (Supplementary 1).

#### 3.3.2. Manifest Refraction

The short-term follow-up at three and six months showed that MRSE was more inclined in the SCXL group (*P* = 0.44 and 0.15), whereas a similar decline of MRSE was found between the both groups at the 12-month visit (*P* = 0.87) (Supplementary 2). For the spherical error, at the early follow-up, both groups have similar improvement (*P* = 0.96), but at the 12-month follow, TECXL seem to have a greater improvement than SCXL (*P* = 0.34) (Supplementary 3). For the cylindrical error, at the three-, six-, and twelve-month follow-up, the decrease was comparable in both groups (*P* = 0.91, 0.76, and 0.56, resp.) (Supplementary 4).

#### 3.3.3. Endothelial Cell Density

Endothelial cell loss was greater with SCXL at three, six, and twelve months (*P* < 0.0001; *P* = 0.13 and 0.009, resp.). However, the change in ECD between the two groups was similar (*P* = 0.69) at 24-month visit (Supplementary 5).

#### 3.3.4. Central Corneal Thickness

A higher early decrease of CCT was stated with SCXL comparing both groups at three, six, and twelve months (*P* = 0.17, 0.04, and 0.06, resp.), even though missing statistical significance at the six- and twelve-month visits. TECXL remain more stable during the the three- to twelve-month follow-up. However, at 24-month visit, an equal reduction of CCT was found in both groups (*P* = 1.00) (Supplementary 6).

### 3.4. Publication Bias

Begg's test (*P* = 0.15, continuity corrected) and Egger's test (*P* = 0.26) were applied, and publication bias of the primary outcome was not significant.

## 4. Discussion

CXL has acquired nowadays popularity for the treatment of progressive keratoconus. SCXL was an epithelial-off procedure: the central corneal epithelial is removed, and riboflavin solution is applied to the exposed corneal stroma. SCXL has been modified over time in favor of a method that does not involve the epithelium debridement, that is, the technique called TECXL. Both techniques (SCXL and TECXL) show great promise for slowing or halting keratoconus in ectatic corneas. However, to our knowledge, this is the first meta-analysis to compare the efficacy and safety of SCXL and TECXL at different follow-up times after surgery, because keratoconus is usually more aggressive in children than adults, which may have different pooling results between the two treatments. So, the meta-analysis only included adult patients (age ≥ 18 years). After our analysis, we found that except for a greater decrease in Kmax with SCXL group, both groups have a comparable effect on the changes in UDVA, CDVA, CCT, and ECD at 24-month follow-up.

Our meta-analysis demonstrated that TECXL group showed a better improvement in UDVA and CDVA at the six- and twelve-month follow-up. For UDVA, TECXL seemed to have a better improvement than SCXL at the 24-month follow-up, but this change might not be clinically meaningful because the WMD (0.09) was small. The study by Bikbova et al. [16] yielded 61.7% of the weight in the meta-analysis because of a larger sample compared with the other study. In terms of CDVA, Wen et al. [14] performed a systematic review and meta-analysis of eight studies, including 455 eyes, and also reported that TECXL seemed to improve CDVA more than SCXL (*P* = 0.14). The outcome is in accordance with the result of our analysis. However, at the 24-month follow-up, our statistical analysis indicated that SCXL showed comparable effects on CDVA compared with TECXL. This might be due to the fact that SCXL provided a great decrease in Kmax between the 12- and 24-month follow-up.

Keratometric change represents progression of keratoconus. The meta-analysis by Meiri et al. [34] showed an improvement in Kmax of −1.00 D at 12 months after SCXL. Uysal et al. [9] also reported a significant decrease in Kmax (*P* < 0.001) at 12 months after SCXL. For TECXL, Aixinjueluo et al. [11] found a significant decrease in Kmax from 59.45 ± 9.34 D at three months (*P* < 0.0001) to 58.11 ± 9.40 D at twelve months (*P* < 0.0001), whereas Ameerh et al. [13] proposed that there was no statistically significant difference in *K*_max_. Our meta-analysis found that TECXL provided less corneal flattening than SCXL regarding maximal keratometry values at the final follow-up. SCXL may be more efficacious in reducing corneal curvature in comparison with TECXL.

For MRSE, this meta-analysis found that both SCXL and TECXL group appear to achieve the same outcomes at the last follow-up. We have found that there is a hyperopic shift in MRSE with both groups, except for one study using TECXL protocol by Stojanovic et al. [32]. In addition, the current meta-analysis found that there is a conflicting result with respect to the MRSE, spherical, and cylindrical error. To calculate the spherical equivalent, measurements of spherical and cylindrical error are used. Thus, we expected similar results comparing these parameters. In contrast, at the six-month follow-up, improvement in MRSE was greater in SCXL, whereas a higher decrease in spherical error was ascertained in the TECXL. The equal decrease in cylindrical error was found in both groups. The conflicting result might be related to the low repeatability of subjective refraction in keratoconus patients by reason of optical irregularities of the distorted cornea causing blurring [35].

Endothelial cell damage may lead to loss of VA on account of corneal edema. Therefore, it is also a clinically important factor influencing patient satisfaction. In our study, TECXL group showed less endothelial cell loss at the three-, six-, and twelve-month visit. However, at the 24-month follow-up, both SCXL and TECXL appear to achieve the same outcomes in ECD. The study by Bikbova et al. [16] yielded 70.2% of the weight in the meta-analysis because of a larger sample size and smaller SD than in the other two studies. In terms of CCT, SCXL group showed more declined CCT than TECXL group at three-, six-, and twelve-month follow-up. Although the statistical difference in CCT at six months was observed, the WMD (8.42 *μ*m) was small, and it is unknown whether this statistically significant difference has an impact on clinical decision-making in the treatment of keratoconus.

This meta-analysis has several limitations that should be considered. First, TECXL group used different composition of riboflavin, concerning different delivery vehicles. Riboflavin in 20% is used by default, but hydroxypropyl methylcellulose (HPMC), ricrolin TE, and iontophoresis (ricrolin+) were also used to allow for a better corneal penetration of riboflavin resulting in a deeper treatment area. HPMC has a drawback, which is the cause of corneal swelling, and thus concentration of stromal bundles decreased, which contributed to the lower efficacy of TECXL. So, the composition and soak time may be influencing factors on the treatment efficacy. Second, several non-RCTs were included to compare SCXL with TECXL, but the NOS score was above seven. Third, in some comparisons, heterogeneity was displayed, possibly due to variation at baseline or missing uniformity in conduct. Finally, only four studies reported the results of 24-month follow-up, and the 24-month data are available at most in 145 eyes (43.7%) of the total eyes with SCXL and 158 eyes (44.1%) of the total eyes with TECXL.

## 5. Conclusions

Based on the current evidence, except for a greater decrease in Kmax with SCXL group, both groups have a comparable effect on visual, pachymetric, and endothelial parameters at 24 months after surgery. Larger studies with a longer follow-up time are necessary to determine whether these techniques are comparable in the long term.

## Figures and Tables

**Table 1:** Characteristics of the included trials.

First author	Year		Country	Study design	Group	Number of eyes	Mean age (year)	Follow-up (months)	UVA irradiation dose (mW/cm^2^) time (min)	UVA cumulative dose (J/cm^2^)	Riboflavin concentration (%)	Riboflavin before irradiation	Riboflavin during irradiation
Akbar [13]	2017		Arabia	Non-RCT	SCXL TECXL	32 32	24.81 24.47	18	3/30 3/30	5.4 5.4	0.1 0.25	With HPMC every 2 min for 20 min With BAC and HPMC one drop every 5s till corneal thickness reached 400u	Every 2 min Every 2 min
Bikbova [15]	2016		Russia	RCT	SCXL TECXL	73 76	30.00 28.00	24	3/30 3/30	5.4 5.4	0.1 0.1	With 20% dextran for 30 min With iontophoresis for 10 min	Every 2 min Every 2 min
Cifariello [14]	2018		Italy	RCT	SCXL TECXL	20 20	24.00 31.00	24	3/30 3/30	5.4 5.4	0.1 0.1	With 20% dextran every 2 min for 30 min With ricrolin TE every 5 min for 30 min	Every 2 min NR
Jouve [20]	2017		France	Non-RCT	SCXL TECXL	40 40	26.2 26.2	24	3/30 10/9	54. 5.4	0.1 0.1	With 20% dextran every 2 min for 20 min With iontophoresis (ricrolin+) for 5 min	Every 5 min —
Kocak [21]	2014		Turkey	Non-RCT	SCXL TECXL	19 17	27.16 27.35	12	3/30 3/30	5.4 5.4	0.1 0.1	With 20% dextran every 2 min for 30 min With ricrolin TE every 2 min for 30 min	Every 2 min Every 2 min
Lombardo a [22]	2016		Italy	RCT	SCXL TECXL	12 22	31.05 29.4	6	3/30 10/9	5.4 5.4	0.1 0.1	With 20% dextran every 3 min for 30 min With iontophoresis (ricrolin+) for 5 min	Every 3 min —
Lombardo b [23]	2017		Italy	RCT	SCXL TECXL	12 22	31.05 29.4	12	3/30 10/9	5.4 5.4	0.1 0.1	With 20% dextran every 3 min for 30 min With iontophoresis (ricrolin+) for 5 min	Every 3 min —
Lombardo c [24]	2019		Italy	RCT	SCXL TECXL	12 22	31.05 29.4	24	3/30 10/9	5.4 5.4	0.1 0.1	With 20% dextran every 3 min for 30 min With iontophoresis (ricrolin+) for 5 min	Every 3 min —
Madeira [25]	2019		Portugal	Non-RCT	SCXL TECXl	10 16	18.69 21.90	12	3/30 6/15	5.4 5.4	0.1	0.1% riboflavin preparation every 2 minutes for 30 minutes 0.25% TE riboflavin preparation every 2 minutes for 20 minutes	Every 5 min Every 5 min
Mastropasqua [26]	2013		Italy	RCT	SCXL TECXL	20 20	23.00 23.00	12	3/30 3/30	5.4 5.4	0.1 0.1	With 20% dextran every 3 min for 15 min With 20% dextran every 3-5 min for 30 min	Every 3 min Every 3 min
Nawaz [27]	2015		India	RCT	SCXL TECXl	20 20	23.95 22.35	6	3/30 3/30	5.4 5.4	0.1 0.1	With 20% dextran every 3–5 min for 30 min With 20% dextran every 3–5 min for 30 min	Every 3–5 min Every 3–5 min
Rossi [28]	2015		Italy	RCT	SCXL TECXL	10 10	30.40 28.00	12	3/30 3/30	5.4 5.4	0.1 0.1	With 20% dextran every 2 min for 30 min With ricrolin TE every 5 min for 30 min	Every 2 min Every 5 min
Rossi a [29]	2018		Italy	RCT	SCXL TECXl	10 10	30.40 27.20	12	3/30 3/30	5.4 5.4	0.1 0.1	With 20% dextran every 2 min for 30 min With ricrolin TE every 5 min for 30 min	Every 2 min Every 5 min
Rossi b [29]	2018		Italy	RCT	SCXL TECXl	10 10	30.40 28.00	12	3/30 10/10	5.4 NR	0.1 0.1	With 20% dextran every 2 min for 30 min With iontophoresis (ricrolin+) for 5 min	Every 2 min Every 2 min
Soeters [18]		2015	Netherlands	RCT	SCXL TECXL	26 35	25.90 26.90	12	3/30 3/30	5.4 5.4	0.1 0.1	With 20% dextran every 2 min for 30 min With ricrolin TE every for 30 min	Every 5 min Every 5 min
Stojanovic [30]		2014	Norway	RCT	SCXL TECXl	20 20	29.5 29.5	12	3/30 3/30	5.4 5.4	0.5 0.5	Vitamin B2 applied alternating every 30 sec BAC and vitamin B2 applied alternating every 30 sec	— —
Vinciguerra [31]		2016	Italy	Non-RCT	SCXL TECXl	20 20	28.2 27.8	12	3/30 10/9	5.4 5.4	0.1 0.1	With 20% dextran every 1 min for 30 min With iontophoresis (ricrolin+) for 5 min	NR NR

SCXL = standard cross-linking, TECXL = transepithelial cross-linking, BAC = benzalkonium chloride, HPMC = hydroxypropyl methylcellulose, RCT = randomized controlled trail, NR = not reported, ricrolin TE = 0.1% riboflavin-15% dextran solution supplement with trihydroxypropyl aminomethane and sodium EDTA, and ricrolin+ = dextran-free ethylenediaminetetraacetic acid and trometamol-enriched 0.1% riboflavin-5-phosphate hypotonic solution; vitamin B^2^ = 0.5% aqueous riboflavin solution without dextran.

**Table 2:** Risk of bias assessment based on the Newcastle-Ottawa Scale for nonrandomized studies.

Item		Akbar et al.	Jouve et al.	Kocak et al.	Madeira et al.	Vinciguerra et al.
Selection	Representativeness of the exposed cohort	1	1	1	1	1
	Selection of the nonexposed cohort	1	1	1	1	1
	Ascertainment of exposure to implants	1	1	1	1	1
	Demonstration that the outcome of interest was not present at the start of the study	0	1	0	0	1

Comparability	Comparability of cohorts on the basis of the design or analysis	1	2	2	2	1

Outcome	Ascertainment of outcome	1	1	1	1	1
	Followed up long enough for outcome to occur	1	1	1	1	1
	Adequacy of follow-up of cohort	1	1	1	1	1

Total score		7	9	8	8	8

## Data Availability

The datasets obtained and/or analyzed during the current study are available from the corresponding author on reasonable request.

## Supplementary Materials

Supplementary Materials Supplementary 1: changes in corrected distance visual acuity in standard corneal cross-linking (SCXL) and transepithelial corneal cross-linking (TECXL). CI = confidence interval; IV = inverse variance. Supplementary 2: changes in mean refractive spherical equivalent in standard corneal cross-linking (SCXL) and transepithelial corneal cross-linking (TECXL). CI = confidence interval; IV = inverse variance. Supplementary 3: changes in spherical error in standard corneal cross-linking (SCXL) and transepithelial corneal cross-linking (TECXL). CI = confidence interval; IV = inverse variance. Supplementary 4: changes in cylindrical error in standard corneal cross-linking (SCXL) and transepithelial corneal cross-linking (TECXL). CI = confidence interval; IV = inverse variance. Supplementary 5: changes in endothelial cells density in standard corneal cross-linking (SCXL) and transepithelial corneal cross-linking (TECXL). CI = confidence interval; IV = inverse variance. Supplementary 6: changes in central corneal thickness in standard corneal cross-linking (SCXL) and transepithelial corneal cross-linking (TECXL). CI = confidence interval; IV = inverse variance.
