# Supplementary figures and images for: Comparison of Standard and Transepithelial Corneal Cross-Linking for the Treatment of Keratoconus: A Meta-analysis
Source: J Ophthalmol. 2021 Jan 29;2021:6679770. doi: 10.1155/2021/6679770 (PMC7864754; doi:10.1155/2021/6679770)

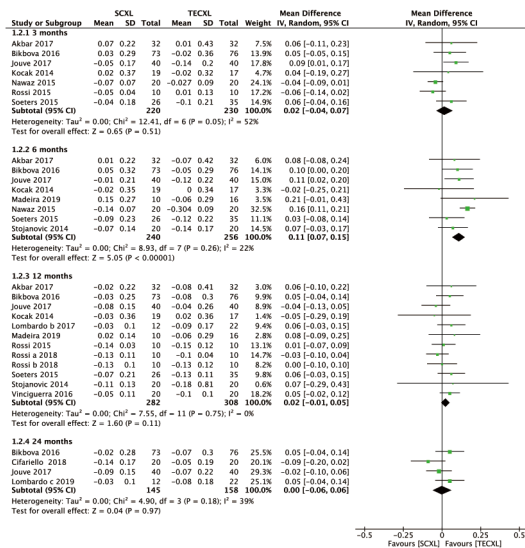

Supplement: Supplementary Materials — Supplementary 1: changes in corrected distance visual acuity in standard corneal cross-linking (SCXL) and transepithelial corneal cross-linking (TECXL). CI = confidence interval; IV = inverse variance. Supplementary 2: changes in mean refractive spherical equivalent in standard corneal cross-linking (SCXL) and transepithelial corneal cross-linking (TECXL). CI = confidence interval; IV = inverse variance. Supplementary 3: changes in spherical error in standard corneal cross-linking (SCXL) and transepithelial corneal cross-linking (TECXL). CI = confidence interval; IV = inverse variance. Supplementary 4: changes in cylindrical error in standard corneal cross-linking (SCXL) and transepithelial corneal cross-linking (TECXL). CI = confidence interval; IV = inverse variance. Supplementary 5: changes in endothelial cells density in standard corneal cross-linking (SCXL) and transepithelial corneal cross-linking (TECXL). CI = confidence interval; IV = inverse variance. Supplementary 6: changes in central corneal thickness in standard corneal cross-linking (SCXL) and transepithelial corneal cross-linking (TECXL). CI = confidence interval; IV = inverse variance. [file 6679770.f1.zip › 6679770.f1/Supplementary 1.pdf]

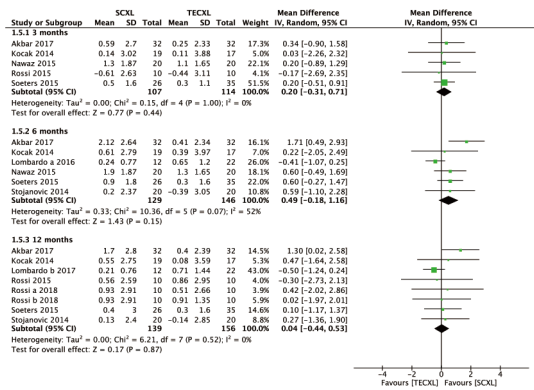

Supplement: Supplementary Materials — Supplementary 1: changes in corrected distance visual acuity in standard corneal cross-linking (SCXL) and transepithelial corneal cross-linking (TECXL). CI = confidence interval; IV = inverse variance. Supplementary 2: changes in mean refractive spherical equivalent in standard corneal cross-linking (SCXL) and transepithelial corneal cross-linking (TECXL). CI = confidence interval; IV = inverse variance. Supplementary 3: changes in spherical error in standard corneal cross-linking (SCXL) and transepithelial corneal cross-linking (TECXL). CI = confidence interval; IV = inverse variance. Supplementary 4: changes in cylindrical error in standard corneal cross-linking (SCXL) and transepithelial corneal cross-linking (TECXL). CI = confidence interval; IV = inverse variance. Supplementary 5: changes in endothelial cells density in standard corneal cross-linking (SCXL) and transepithelial corneal cross-linking (TECXL). CI = confidence interval; IV = inverse variance. Supplementary 6: changes in central corneal thickness in standard corneal cross-linking (SCXL) and transepithelial corneal cross-linking (TECXL). CI = confidence interval; IV = inverse variance. [file 6679770.f1.zip › 6679770.f1/Supplementary 2.pdf]

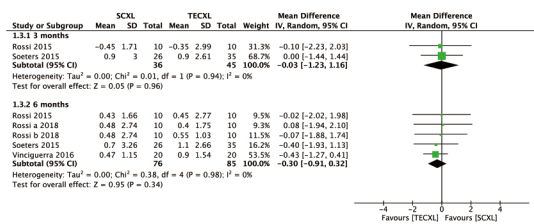

Supplement: Supplementary Materials — Supplementary 1: changes in corrected distance visual acuity in standard corneal cross-linking (SCXL) and transepithelial corneal cross-linking (TECXL). CI = confidence interval; IV = inverse variance. Supplementary 2: changes in mean refractive spherical equivalent in standard corneal cross-linking (SCXL) and transepithelial corneal cross-linking (TECXL). CI = confidence interval; IV = inverse variance. Supplementary 3: changes in spherical error in standard corneal cross-linking (SCXL) and transepithelial corneal cross-linking (TECXL). CI = confidence interval; IV = inverse variance. Supplementary 4: changes in cylindrical error in standard corneal cross-linking (SCXL) and transepithelial corneal cross-linking (TECXL). CI = confidence interval; IV = inverse variance. Supplementary 5: changes in endothelial cells density in standard corneal cross-linking (SCXL) and transepithelial corneal cross-linking (TECXL). CI = confidence interval; IV = inverse variance. Supplementary 6: changes in central corneal thickness in standard corneal cross-linking (SCXL) and transepithelial corneal cross-linking (TECXL). CI = confidence interval; IV = inverse variance. [file 6679770.f1.zip › 6679770.f1/Supplementary 3.pdf]

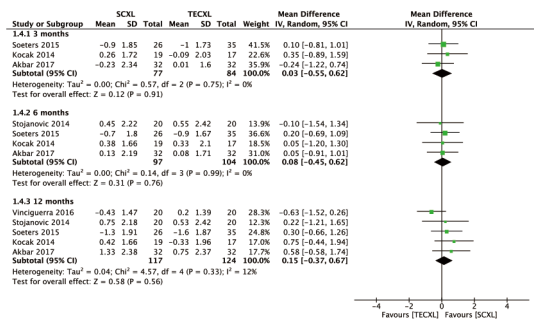

Supplement: Supplementary Materials — Supplementary 1: changes in corrected distance visual acuity in standard corneal cross-linking (SCXL) and transepithelial corneal cross-linking (TECXL). CI = confidence interval; IV = inverse variance. Supplementary 2: changes in mean refractive spherical equivalent in standard corneal cross-linking (SCXL) and transepithelial corneal cross-linking (TECXL). CI = confidence interval; IV = inverse variance. Supplementary 3: changes in spherical error in standard corneal cross-linking (SCXL) and transepithelial corneal cross-linking (TECXL). CI = confidence interval; IV = inverse variance. Supplementary 4: changes in cylindrical error in standard corneal cross-linking (SCXL) and transepithelial corneal cross-linking (TECXL). CI = confidence interval; IV = inverse variance. Supplementary 5: changes in endothelial cells density in standard corneal cross-linking (SCXL) and transepithelial corneal cross-linking (TECXL). CI = confidence interval; IV = inverse variance. Supplementary 6: changes in central corneal thickness in standard corneal cross-linking (SCXL) and transepithelial corneal cross-linking (TECXL). CI = confidence interval; IV = inverse variance. [file 6679770.f1.zip › 6679770.f1/Supplementary 4.pdf]

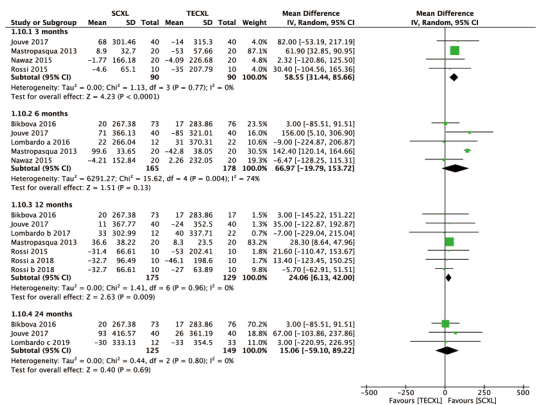

Supplement: Supplementary Materials — Supplementary 1: changes in corrected distance visual acuity in standard corneal cross-linking (SCXL) and transepithelial corneal cross-linking (TECXL). CI = confidence interval; IV = inverse variance. Supplementary 2: changes in mean refractive spherical equivalent in standard corneal cross-linking (SCXL) and transepithelial corneal cross-linking (TECXL). CI = confidence interval; IV = inverse variance. Supplementary 3: changes in spherical error in standard corneal cross-linking (SCXL) and transepithelial corneal cross-linking (TECXL). CI = confidence interval; IV = inverse variance. Supplementary 4: changes in cylindrical error in standard corneal cross-linking (SCXL) and transepithelial corneal cross-linking (TECXL). CI = confidence interval; IV = inverse variance. Supplementary 5: changes in endothelial cells density in standard corneal cross-linking (SCXL) and transepithelial corneal cross-linking (TECXL). CI = confidence interval; IV = inverse variance. Supplementary 6: changes in central corneal thickness in standard corneal cross-linking (SCXL) and transepithelial corneal cross-linking (TECXL). CI = confidence interval; IV = inverse variance. [file 6679770.f1.zip › 6679770.f1/Supplementary 5.pdf]

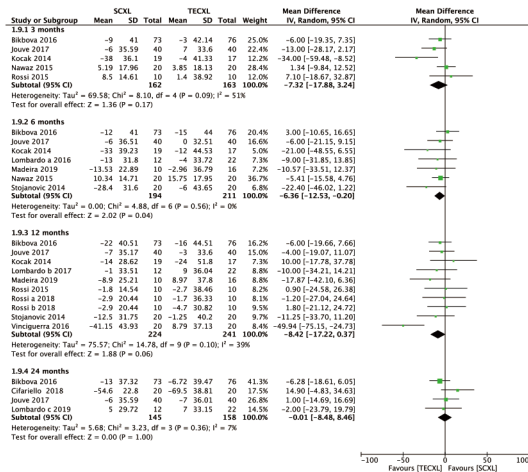

Supplement: Supplementary Materials — Supplementary 1: changes in corrected distance visual acuity in standard corneal cross-linking (SCXL) and transepithelial corneal cross-linking (TECXL). CI = confidence interval; IV = inverse variance. Supplementary 2: changes in mean refractive spherical equivalent in standard corneal cross-linking (SCXL) and transepithelial corneal cross-linking (TECXL). CI = confidence interval; IV = inverse variance. Supplementary 3: changes in spherical error in standard corneal cross-linking (SCXL) and transepithelial corneal cross-linking (TECXL). CI = confidence interval; IV = inverse variance. Supplementary 4: changes in cylindrical error in standard corneal cross-linking (SCXL) and transepithelial corneal cross-linking (TECXL). CI = confidence interval; IV = inverse variance. Supplementary 5: changes in endothelial cells density in standard corneal cross-linking (SCXL) and transepithelial corneal cross-linking (TECXL). CI = confidence interval; IV = inverse variance. Supplementary 6: changes in central corneal thickness in standard corneal cross-linking (SCXL) and transepithelial corneal cross-linking (TECXL). CI = confidence interval; IV = inverse variance. [file 6679770.f1.zip › 6679770.f1/Supplementary 6.pdf]
